# World Mental Health Day 2023: We must leave no one behind in the response to HIV and mental health

**DOI:** 10.1002/jia2.26183

**Published:** 2023-10-10

**Authors:** Milton L. Wainberg, George Gustaaf Wolvaardt, Lidia Gouveia, Erin Ferenchick

**Affiliations:** ^1^ Columbia University – Department of Psychiatry New York NY USA; ^2^ Foundation for Professional Development Pretoria South Africa; ^3^ Department of Mental Health Ministry of Health Maputo Mozambique; ^4^ Center for Family and Community Medicine Columbia University New York NY USA

1

Despite great achievements in the response to HIV over the past four decades, emerging data are alarming. They reveal that progress is faltering with only a 3.5% drop in global acquisitions from 2020 to 2021 ‐ the smallest annual decline since 2016.^1^ The COVID‐19 pandemic and growing humanitarian and climate crises have challenged health systems globally, profoundly impacting the HIV response and threatening the achievement of the United Nations 95‐95‐95 targets. There has been considerable debate about what efforts are needed if we are to get back on track. To commemorate World Mental Health Day, we advocate that part of the solution must ensure sustained political and financial commitment to scale‐up evidence‐based interventions addressing the mental health needs of people living with, at risk of, or affected by HIV.

Mental health conditions are common in people living with HIV (PLHIV). They occur as risk factors for HIV, coincidentally with HIV, or as a result of HIV acquisition and its complications. Multiple studies from Africa have estimated that the prevalence of mental health conditions in PLHIV range from 19%^2^ to about 50%,^3^ with the prevalence of depression estimated to be 24%, compared with less than 3% for the general population.^4^ Similar results have been found in other parts of the world (e.g., India,^5^ China,^6^ the US and Canada^7^), especially among key populations (e.g., transgender persons),^8^ and people with mental illness have higher rates of HIV globally.^7^ We now recognize that the coexistence of mental health conditions leads to poor outcomes along the HIV care continuum,^7^ and depression has been identified as one of the strongest predictors of poor ART adherence.^9^, ^10^ Yet, the availability of screening and treatment for mental health conditions in HIV clinics in many parts of the world remains limited.^11^

The political landscape, however, seems to be shifting. At the global level, WHO and UNAIDS recently released new guidance on the integration of mental health services into HIV programming.^1^, ^12^, ^13^ This has been accompanied by a commitment to mental health in the 2023–2028 Strategy of the Global Fund to Fight HIV/AIDS, Tuberculosis and Malaria, and PEPFAR's recent commentary^14^ outlines their next steps for addressing mental health programming gaps for PLHIV. At the country level, an increasing number of national strategic plans for HIV ‐ such as in Kenya, Uganda and South Africa ‐ now include mental health as part of their strategic priorities. We also have heard the powerful voice of civil society begin to mobilize around mental health.^15^

Importantly, this momentum builds upon ongoing efforts to strengthen the evidence in support of mental health interventions for PLHIV. For example, research in Mozambique has focused on training community health workers to use a locally validated digital tool called the Electronic Mental Wellness Tool (e‐mwTool) to screen, diagnose and treat common mental and substance use disorders among people living with, at risk of, or affected by HIV, and refer those with more serious conditions to trained primary care providers, supervised by psychiatric technicians who are the mental health workforce of the country. Through this work, we have seen the potential of digitally enhanced community‐led interventions. The e‐mwTool leverages technology‐based community screening to detect and provide measurement‐based care for mental disorders using GPS coordinates, supports facility‐ and community‐level implementation of task‐shared evidence‐based interventions with rigour and tracks intervention implementation and clinical outcomes.^16^, ^17^, ^18^ The e‐mwTool has global relevance and has been validated and is being implemented in other settings as well.^19^, ^20^ A research training program in Cambodia, Malaysia, the Philippines and Thailand, nested in the International Epidemiology Databases to Evaluate AIDS (IeDEA Asia‐Pacific), is building local and regional capacity to effectively implement integrated HIV‐mental health care strategies using implementation science methods, supporting the adaptation of the e‐mwTool to the region. Ongoing research in South Africa highlights another example of collaborative work aimed at understanding the effectiveness of mental health services within integrated care. Through a PEPFAR‐U.S. Centers for Disease Control and Prevention‐funded initiative “Improving Mental Health and HIV/TB Service Integration,” efforts are being scaled‐up to develop capacity for community screening, diagnosis, and counselling of common mental health conditions with the concurrent mapping of referral pathways within and between public and civil society sectors. This is the first integration initiative at scale in South Africa and has gained the attention of the South African Government. This has translated into an increased commitment to mental health, as evidenced by the robust inclusion of mental health in their updated National Strategic Plan for HIV, TB and STIs 2023–2028.^21^

Despite a strong commitment to research and early signs of a shifting landscape, challenges remain. Until we are able to foster the same level of political and financial commitment for treating depression, anxiety, and substance abuse for PLHIV as we have committed to ensuring equitable access to antiretroviral medicines, we will continue to leave people behind in the HIV response. On average, countries dedicate less than 2% of their healthcare budgets to mental health,^9^ and there is minimal international developmental assistance allocated for mental health overall. Securing both the necessary political will and adequate financial resources to deliver evidence‐based mental health services at the speed and scale required for impact must be a shared priority with shared accountability. A potential positive development, for example, would be if the *Mental Health in International Development and Humanitarian Settings (MINDS) Act*,^22^ which was reintroduced in early March 2023, were passed by the U.S. Congress. This legislation would mainstream mental health and psychosocial support in U.S. government‐funded international assistance, making foreign aid programs more effective by supporting best practices in the field of global mental health and laying the groundwork for improving health outcomes overall. In addition to a global call for action through the *MINDS Act*, attention must be also paid to national and subnational health sector planning and budgeting processes, as well as governance and leadership issues. Appropriate departments and ministries (e.g., Mental Health, HIV and/or Primary Care) should be engaged in this work so that activities are well planned and implemented for the successful scale‐up of evidence‐based prevention and treatment of coexisting mental health conditions for PLHIV.

As we reflect on the theme for World Mental Health Day this year, “Mental Health is a human right,” let us work together to ensure that those living with, at risk of, or affected by HIV have the right to the highest attainable standard of physical and mental health. We must leave no one behind.

## COMPETING INTERESTS

No conflict of interests for any of the authors.

## AUTHORS’ CONTRIBUTIONS

MLW, GGW, LG and EF contributed to the writing of the manuscript.

## FUNDING

NIH ‐ D43TW009675; U19MH113203; R01AA025947; D43TW011302; T32MH096724; and U01AI069907, SAMHSA ‐ H79FG00075, CDC ‐ GH002393

## DISCLAIMER

None to report
